# Key recommendations from the 2021 “inclusion of older adults in clinical research” workshop

**DOI:** 10.1017/cts.2022.1

**Published:** 2022-01-07

**Authors:** Darina V. Petrovsky, Lan N. Ðoàn, Maria Loizos, Rachel O’Conor, Micah Prochaska, Mazie Tsang, Rachel Hopman-Droste, Tara C. Klinedinst, Aarti Mathur, Karen Bandeen-Roche, Odette van der Willik, Stephen B. Kritchevsky

**Affiliations:** 1 Rutgers University, New Brunswick, NJ, USA; 2 New York University Grossman School of Medicine, New York, NY, USA; 3 Icahn School of Medicine at Mount Sinai, New York, NY, USA; 4 Northwestern University, Evanston, IL, USA; 5 University of Chicago, Chicago, IL, USA; 6 University of California, San Francisco, CA, USA; 7 Pearson, London, UK; 8 University of Pittsburgh, Pittsburgh, PA, USA; 9 Johns Hopkins University, Baltimore, MD, USA; 10 American Federation for Aging Research, New York, NY, USA; 11 Wake Forest School of Medicine, Winston-Salem, NC, USA

**Keywords:** Clinical trials, older adults, inclusion

## Abstract

Older adults are often underrepresented in clinical research, even though older adults are major consumers of novel therapies. We present major themes and recommendations from the 2021 "Inclusion of Older Adults in Clinical Research" Workshop, convened by the Clinical and Translational Science Award (CTSA) Inclusion of Older Adults as a Model for Special Populations Workgroup and the Research Centers Collaborative Network (RCCN). The goal of this workshop was to develop strategies to assist the research community in increasing the inclusion of older adults in clinical research. Major identified barriers include historical lack of federal guidelines, ageist biases and stereotypes, and lack of recruitment and retention techniques or infrastructure focused on older adults. Three key recommendations emerged: 1) engaging with the policymaking process to further promote inclusion; 2) using the CTSA Workgroup Presentation Materials Library and other resources to overcome ageism, and 3) building institutional capacity to support age inclusion.

## Introduction

Over the last century, there has been great progress in life expectancy in the USA. However, this change in longevity is accompanied by increases in the burden of multimorbidity, polypharmacy, and frailty. Despite the growth in the number of medically complex older adults, many clinical trials exclude such individuals, and as a result, older adults are underrepresented in clinical research [1,2].

Recognition of the need to address underrepresentation of older adults in clinical research has grown in recent years [3,4]. The Clinical and Translational Science Award (CTSA) Inclusion of Older Adults as a Model for Special Populations Workgroup in partnership with the Research Centers Collaborative Network (RCCN) convened the “Inclusion of Older Adults in Clinical Research” Workshop in February 2021 (herein referred to as the Workshop). The Workshop sought to convene cross-disciplinary perspectives, including key thought leaders from National Institute on Aging (NIA) center programs (Appendix Table S1 lists all workshop speakers). This manuscript presents summaries of discussion from the Workshop to inform the inclusion of older adults in clinical research.

## NIH Inclusion Across the Lifespan

The National Institutes of Health (NIH) is the largest funder of research in the US; therefore, NIH policies have significant impact on research practices. The NIH began setting policies to stimulate broader inclusion in 1986, with the policy encouraging researchers to include women in studies. In 2017, the NIH “Inclusion Across the Lifespan” Workshop hosted a meeting to discuss the representation of older adults (65 years and older) in research [5]. In response to the 21st Century Cures Act, which directed the NIH to ensure inclusion of a broad range of participants in research [6], the NIH updated their policy and guidelines to include all ages in clinical research, unless there were specific and compelling scientific or ethical justifications for participant age restrictions [3,7].

Furthermore, in 2015 the CTSA Program established the Hub Research Capacity Cores and instituted the LIFESPAN Domain Task Force as one of five major platforms for network-wide communication and collaboration dedicated to promoting age inclusivity in research. This led to the development of an “Inclusion of Older Adults as a Model for Special Populations” working group (henceforth, “CTSA Inclusion Workgroup”) in 2019 with the mission of increasing inclusion of older adults in research.

In 2020, the NIH convened a second workshop “Inclusion Across the Lifespan” that examined the representation of racial/ethnic minority and other marginalized older populations in clinical studies [8]. This workshop led to recommendations on adjusting study inclusion and exclusion criteria to represent target populations, being age-inclusive in trial design and metrics, utilizing best practices and completing relevant training to recruit and retain older adults in studies, and identifying social determinants of health across age groups. The NIH has continued these efforts to ensure older adults are included in clinical research to represent target populations.

## State of the Inclusion of Older Adults

The Workshop was organized to discuss the current state of older adult inclusion, overall and among older adults of color with panelists featuring oncology, cardiology, Alzheimer’s Disease and Related Dementias (ADRD) as examples. In cancer research, clinical trials often do not include older adults, which limits data on tolerability, leaving clinicians to improvise dose adjustments in older adults. Researchers in geriatric oncology are focused on increasing the inclusion of older adults in clinical trials and optimizing the screening and management of older adults with cancer. When examining data on 224,766 older adults with cancer supporting 105 Federal Drug Administration (FDA) drug applications according to age distributions of <65, 65–69, 70–74, 75–79, and ≥80, the participation rates of older adults with cancer in clinical trials were significantly lower compared to corresponding participation rates in the US cancer population. For example, 4% of all clinical trial participants enrolled between 2005 and 2015 were over the age of 80, whereas approximately 16% of older adults age 80+ in 2013 were diagnosed with cancer [9]. This led to the American Society of Clinical Oncology recommendation in 2018 that oncologists consider performing geriatric assessments in older adults who receive chemotherapy to screen for cognitive impairment or impaired nutrition [10].

Similarly, older adults with cardiovascular disease are frequently excluded from cardiovascular clinical trials, despite the growing number of older adults with these diseases [11,12]. More recently, however, cardiovascular clinicians and researchers shifted from thinking of the heart as the epicenter to a more integrated view that includes functional outcomes such as frailty and cognitive impairment. Future challenges facing cardiovascular research include selection of meaningful and measurable clinical outcomes and logistics of conducting research with older adults.

Older adults with ADRD continue to be frequently excluded from clinical research. In a 2012 review, 16% of studies applied recruitment methods that were likely to reduce participation of older adults living with ADRD and only 6% of studies provided justification for exclusion [13]. A recent analysis of Medicare beneficiaries with ADRD concluded that inclusion criteria in clinical trials of Aducanumab, a drug aimed at slowing down Alzheimer’s disease progression, would have excluded more than 92% of eligible older adults [14]. Research opportunities for persons living with ADRD include research on new models of decision-making, determining preferences, sources of exclusion, training needs, and science of engagement [15].

Lastly, health disparities cut across all chronic diseases and contribute to ageism. Ageism is defined as “negative or positive stereotypes, prejudice and/or discrimination against (or to advantage of) elderly people on the basis of their chronological age…” [16]. Potentially stemming from ageism, health disparities are differences in health that exist across disparate groups, including race, ethnicity, and age [17]. Older racial/ethnic minority adults, who face significant social and economic inequities that contributes to their mistrust of the medical system, are less likely to participate in clinical research [18]. To mitigate this, researchers should conduct research with the lens of cultural humility, develop culturally and linguistically concordant interventions, and build trust by learning from older adults’ lived experiences. Many NIA-funded efforts are underway to examine diversity, recruitment, and retention in aging research.

## Barriers to the Inclusion of Older Adults

Workshop panelists identified three main barriers to the inclusion of older adults in clinical trials: 1) historical lack of federal guidelines; 2) ageism; and 3) lack of recruitment and retention techniques/infrastructure focused on older adults.

The recent changes to NIH’s inclusion policy may have a substantial impact on the inclusion of older adults with researchers having to justify age limits for inclusion criteria in grant applications. However, disease-based exclusions will still differentially impact older adults. In 2020, the FDA published guidance to broaden the eligibility criteria to increase the diversity of study participants with comorbidities (e.g., sex, race, ethnicity, age, location of residency) [19]. This guidance recommended increasing inclusion of older adults by using adaptive clinical trial designs and during early clinical development (e.g., describing differences in drug metabolism and clearance across populations). A high priority will be to evaluate the effect of these changes on age inclusion to determine if additional action on the part of funding and regulating bodies is necessary.

Ageism can play out through the entire human research subject protections process: study design, institutional review board (IRB) evaluation, and conduct of research. Contributing to implicit bias, benevolent prejudice refers to a “superficially positive type of prejudice… expressed in terms of positive beliefs and emotional responses, which results in keeping the group of members experiencing prejudice in inferior positions in society” [20]. Older adults, for example, may be seen as needing care and concern, supporting the notion that they require greater protection in and from research. Implicit biases and assumptions about what older adults can and cannot do may impede their inclusion. Implicit bias could be addressed by educating IRB members, researchers, and staff of older adults’ meaningful contributions to research.

Successful recruitment strategies used in population-based survey research may provide useful techniques to increase representation of older adults in clinical research outside of a traditional clinical setting. Examples of survey research include surveys supported by the NIH (e.g., Health and Retirement Survey), Census Bureau (e.g., American Community Survey), and National Center for Health Statistics (e.g., National Health Interview Survey). Surveys can provide nuanced, longitudinal information on the national trends in health and social determinants of health in representative samples of older adults. Lessons on inclusion of older adults in population-based surveys include using multi-pronged recruitment models, building relationships and trust with older adults by engaging with them outside of clinic visits, using financial incentives, and engaging with care partners. In addition, some older adults may need assistance in getting transportation to research sites or may need help navigating complicated medical facilities. Centralized infrastructure support would make it easier for investigators to access the resources required to promote inclusion.

## Making the Case

In order to make the case to include older adults in research, schema such as the 5Ts framework (Target Population, Team, Tools, Time and Tips to Accommodate) can guide researchers to support inclusion of older adults in studies [21]. Below we outline additional arguments identified during the Workshop.

First, inclusion of older adults is consistent with the ethical value and principle of justice, which is defined by The Belmont Report as risk and benefits of research that are equally shared amongst each person [22]. Second, inclusion aligns with the scientific values of generalizability and reproducibility. Older adults constitute a large proportion of end-users for novel therapies. It is, therefore, critical that results from clinical research are generalizable to this population. The only way to understand if findings are reproducible across the lifespan is by inclusion across the lifespan. Third, there are practical benefits of including older adults in research. The inclusion of higher-risk older adults can increase study power for certain clinical events for an equivalent sample size. Furthermore, older adults may have more flexible schedules compared to younger adults and view participating in research as leaving a legacy for the next generation [23]. These practical advantages can translate to more rigorous and impactful clinical trials.

## Anticipating and Adapting to Possible Effects of Increased Age Inclusion

Concerns about selective participation and study dropout leading to decreased sample size and bias related to misrepresentation of the target population may contribute to the exclusion of older adults. As a result, study treatments generalize to younger, healthier participants as opposed to the intended target. However, in the experience of the Workshop participants, retention of older adults is usually excellent. Nevertheless, at the design stage, sample sizes can be calculated to account for incomplete retention, and collection of auxiliary data related to dropout can mitigate biases. If researchers are concerned about missed measures, prioritizing key measures early in the assessment can help protect the validity of data. Stereotypes related to the cognitive capacity of older adults may engender concerns about measurement accuracy. Design choices can mitigate these concerns. For example, wearable sensors can collect data on exposures and outcomes and can overcome concerns about the ability of older adults with cognitive and physical impairments. In longer-term studies, competing risks, such as death prior to achieving a study outcome, can be managed and even leveraged to additional insight using appropriate statistical methods.

Lastly, researchers may have concerns about treatment heterogeneity across age groups as well as heterogeneity in older adult health. In some cases, these can be leveraged for increased power in estimating the relationships between independent and dependent variables [24]. Both designs (e.g., adaptive) and analytic methods are available to compare treatment effects in subgroups in a principled way [25]. Including older adults in clinical research may introduce complexity for data analysis and interpretation of findings, and we recommend engaging a statistician or an epidemiologist to assist in the development of design and analysis strategies for overcoming these barriers.

## Recommendations

The key takeaway from this Workshop was that inclusion of older adults is paramount to increase the reproducibility and generalizability of research findings. Older adults continue to be underrepresented in clinical research, despite being one of the largest consumers for therapeutics and medical interventions. Key recommendations to promote the inclusion of older adults in clinical research include the following: 1) engaging with the policymaking process to further promote inclusion; 2) using the CTSA Presentation Materials Library on Inclusion of Older Adults in Clinical and Translational Research (https://clic-ctsa.org/education/kits/presentation-materials-library-inclusion-older-adults-clinical-and-translational) and other resources to overcome ageism; and 3) building institutional capacity to support age inclusion (Table 1).

**Table 1.** Key themes and recommendations

Barriers	Recommendations
Historical lack of federal guidelines and policies	• Introduce fellow colleagues and the public to the National Institutes of Health and the United States Food and Drug Administration policies for inclusion of older adults in research • Advocate for older adults in front of stakeholder groups and agencies • Raise awareness of the lack of inclusion • Highlight the stories and individual experiences of older adults when engaging with policymakers • Advocate for eliminating strict exclusion criteria of older adults based on arbitrary risk assessment by Institutional Review Boards (IRBs) and funding agencies
Ageism and negative stereotypes about older adults	• Equip yourself, IRB members, researchers, and staff with knowledge about what older adults can contribute to clinical research using the toolkit • Build trust by learning from older adults’ lived experiences • Seek training in cultural practices across diverse groups of older adults
Lack of age-friendly initiatives in recruitment and retention	• Invest in recruitment and retention efforts • Make study participation easier for older adults by providing incentives and travel reimbursement • Promote a physically accessible clinical research environment • Include relevant and meaningful clinical outcomes to older adults (i.e., function, quality of life) • Set up a realistic budget • Make study materials available in other languages, besides English • Work with a statistician and/or epidemiologist to address special statistical considerations and subgroup analyses • Educate IRBs on the importance of assessing and addressing ageism

When engaging in policy efforts, researchers should introduce others to the NIH and the FDA policies for inclusion of older adults and be vocal in their support of new policies to others. In addition, reviewers in study sections may need to be trained on identifying poor justification for exclusion of older adults in grant applications. Advocacy efforts should include presentations and conversations with stakeholder groups, elected officials, policymakers, and agencies on the importance of inclusion of older adults in research. IRBs should eliminate strict exclusion criteria for older adults based on arbitrary risk assessment. Personal stories matter and may be more effective than statistics in motivating change – researchers can use these stories to engage with policymakers and IRBs.

Barriers for older adult participation in research include lack of resources or limited physical capacity. Institutions can support core services to mitigate these barriers by providing transportation and navigation for older adults who need such services. Given persistent lack of trust in the medical community, researchers should build trust with the research participants by learning from older adults’ lived experiences. As a result, researchers will be better able to promote inclusive practices and develop better research processes that protect older adults. Age-friendly movements focused on health systems, cities, and universities and the Reframing Aging Initiative are examples of strategies to create age-inclusive communities.

The lack of age-friendly initiatives in recruitment and retention efforts contribute to the exclusion of older adults in research. We encourage researchers to involve older adults in all phases of the research cycle and make justifications for inclusion of older adults in research clear. Older adults need to be given an opportunity to contribute and understand the science underlying research. This can be accomplished by creating more opportunities for older adult representation in a citizen science or professional researcher program, like the Professional Research Assistant program at the University of Colorado. Quality improvement strategies like rapid cycles can be helpful in testing and identifying most successful recruitment strategies. In addition, implementation outcomes can be incorporated into study design to evaluate intervention success [26,27]. This approach promotes the dissemination of successful strategies to the broader research community.

The CTSA Inclusion Workgroup under the NIH National Center for Advancing Translational Sciences (NCATS) is a recent effort aimed at equipping research teams with tools to facilitate successful inclusion of diverse older adults in clinical research. It has provided a slide set repository focused on ways to improve recruitment, enrollment, and retention of older adults, particularly from marginalized backgrounds and underresourced settings (https://clic-ctsa.org/education/kits/presentation-materials-library-inclusion-older-adults-clinical-and-translational). In addition, slide set modules provide examples of successful recruitment approaches, methods to address typical reasons older adults are excluded from research, ways to adapt studies, and ethical considerations regarding consent of older adults with limited decision-making capacity.

Building institutional capacity to support age inclusion will require solid infrastructure support, resources, and designated funds. For example, having a center that supports age-friendly recruitment and retention would allow researchers access to resources and toolkits to promote inclusion in clinical research. Resources may include webinars with stakeholders engaged in aging research (e.g., funders, academic researchers, community leaders, and coalitions), peer-to-peer conversations, and continuing education opportunities that keep researchers up to date with federal policies on inclusion. Other educational topics may include ways to combat ageism, how to actively engage and build trust with diverse older adult populations, how to address IRB-related challenges related to including older adults in research, approaches to developing and implementing age-friendly initiatives in recruitment and retention. At a minimum, institutions could launch pilot grants focused on research on older adult populations to support a pipeline of aging-related research among scientists.

## Conclusion

The aim of the Workshop, a collaboration between the CTSA Inclusion Workgroup and RCCN, was to develop and disseminate strategies to assist the research community with improving the inclusion of older adults in clinical research. Researchers should engage with the policymaking process to further promote inclusion, include the voice of older participants in every stage of research, and promote age-friendly initiatives in their institutions to make a significant impact on the health of older adults, who are one of the fastest-growing populations in the U.S.

## Supplementary material

For supplementary material accompanying this paper visit https://doi.org/10.1017/cts.2022.1. click here to view supplementary material
