# Supplementary material for: Key recommendations from the 2021 “inclusion of older adults in clinical research” workshop
Source: J Clin Transl Sci. 2022 Jan 7;6(1):e55. doi: 10.1017/cts.2022.1 (PMC9161040; doi:10.1017/cts.2022.1)
Supplement: Supplementary file 1 [file S2059866122000012sup001.docx]

**Supplementary Material**

**Table S1: Workshop Panelists**

| **Name** | **Affiliation** |
| --- | --- |
| Amanda Sonnega | University of Michigan |
| Ana Iltis | Wake Forest University |
| Andrea Gilmore-Bykovskyi | University of Wisconsin-Madison |
| Barbara Radziszewska | National Institute on Aging |
| Christine Ritchie | Harvard Medical School |
| Dan Forman | University of Pittsburgh |
| David Marquez | University of Illinois at Chicago |
| David Weir | University of Michigan |
| Elizabeth Eckstrom | Oregon Health and Science University |
| Jay Magaziner | University of Maryland |
| Jerry Gurwitz | University of Massachusetts |
| Karen Bandeen-Roche | Johns Hopkins University |
| Marie Bernard | National Institute on Aging |
| Mark Supiano | University of Utah |
| Michael Wasserman | California Association of Long Term Care Medicine |
| S.W. Johnny Lau | Food and Drug Administration |
| Stephen Kritchevsky | Wake Forest School of Medicine |
| Supriya Mohile | University of Rochester |
| Wendy Kohrt | University of Colorado, Denver |
